# Lung Ultrasound, Clinical and Analytic Scoring Systems as Prognostic Tools in SARS-CoV-2 Pneumonia: A Validating Cohort

**DOI:** 10.3390/diagnostics11122211

**Published:** 2021-11-26

**Authors:** Jaime Gil-Rodríguez, Michel Martos-Ruiz, José-Antonio Peregrina-Rivas, Pablo Aranda-Laserna, Alberto Benavente-Fernández, Juan Melchor, Emilio Guirao-Arrabal

**Affiliations:** 1 Internal Medicine Unit, San Cecilio University Hospital, 18012 Granada, Spain; jaigilro@gmail.com (J.G.-R.); michelmartosruiz@gmail.com (M.M.-R.); pablo.aranlas@gmail.com (P.A.-L.); alberto.benavente.sspa@juntadeandalucia.es (A.B.-F.); 2 Infectious Diseases Unit, San Cecilio University Hospital, 18012 Granada, Spain; joseperegrina91@gmail.com; 3 Department of Statistics and Operations Research, University of Granada, 18011 Granada, Spain; 4 Biomechanics Group (TEC-12), Instituto de Investigación Biosanitaria (IBS), 18012 Granada, Spain; 5 Research Unit “Modelling Nature” (MNat), University of Granada, 18011 Granada, Spain

**Keywords:** SARS-CoV-2, COVID-19, lung, ultrasound, early warning score

## Abstract

At the moment, several COVID-19 scoring systems have been developed. It is necessary to determine which one better predicts a poor outcome of the disease. We conducted a single-center prospective cohort study to validate four COVID-19 prognosis scores in adult patients with confirmed infection at ward. These are National Early Warning Score (NEWS) 2, Lung Ultrasound Score (LUS), COVID-19 Worsening Score (COWS), and Spanish Society of Infectious Diseases and Clinical Microbiology score (SEIMC Score). Our outcomes were the combined variable “poor outcome” (non-invasive mechanical ventilation, intubation, intensive care unit admission, and death at 28 days) and death at 28 days. Scores were analysed using univariate logistic regression models, receiver operating characteristic curves, and areas under the curve. Eighty-one patients were included, from which 21 had a poor outcome, and 9 died. We found a statistically significant correlation between poor outcome and NEWS2, LUS > 15, and COWS. Death at 28 days was statistically correlated with NEWS2 and SEIMC Score although COWS also performs well. NEWS2, LUS, and COWS accurately predict poor outcome; and NEWS2, SEIMC Score, and COWS are useful for anticipating death at 28 days. Lung ultrasound is a diagnostic tool that should be included in COVID-19 patients evaluation.

## 1. Introduction

Pneumonia caused by SARS-CoV-2 (COVID-19) has been an unprecedented health disruption in recent decades. In December 2019, a lung disease outbreak due to a previously unknown pathogen was announced in the city of Wuhan, China. Since then, according to the Coronavirus Resource Center at Johns Hopkins University, as of 1 June 2021, more than 170 million cases and 3.5 million deaths have been reported all around the world [1]. Of these data, more than 50 million cases correspond to Europe and, in particular, more than 3.5 million to Spain [2]. This has resulted in 250,000 hospital admissions in Spain, from which 25,000 went to intensive care units [3].

One of the difficulties in assessment and management lies on knowing which patients will have a more unfavourable evolution. Acute respiratory distress syndrome (ARDS) is the worse consequence of SARS-CoV-2 inflammatory process in the lungs, needing frequent high-flow nasal oxygen therapy or noninvasive mechanical ventilation (NIMV), invasive mechanical ventilation (IMV), or extracorporeal membrane oxygenation (ECMO) in critical cases [4]. This leads to an overuse of intensive care unit resources, extended hospital stays, and a high mortality rate of this population.

Several scoring systems have tried to classify patients at admission [5,6,7,8,9]. Classifying patients has the utility of trying to predict which of them may have a worse outcome in order to provide them an earlier administration of ventilatory support or drugs that change the patient’s prognosis. These scores are usually based on clinical and/or analytical data.

However, lung ultrasound has been reported to be a promising tool for the diagnosis and follow-up of pneumonia, interstitial diseases, and ARDS [10,11]. Lung damage originated from COVID-19 is typically patchy and subpleural, which makes COVID-19 specially interesting to be evaluated by lung ultrasound [12]. Its role in the pandemic has so far been secondary, but it has proven to be a useful tool for the stratification of severity and risk of death in these patients [13], as supported by a strong correlation between lung ultrasound and CT, with greater accessibility and speed at lower cost [14].

The aim of our study is to evaluate some of these scoring systems in order to validate them and assess which one of them better predicts a worse outcome in our cohort.

## 2. Materials and Methods

### 2.1. Study Design and Setting

We conducted a single-center prospective cohort study to validate four COVID-19 prognosis scores. The study protocol was approved by the regional ethics committee with the code 0259-N-21. This study was written in accordance with the TRIPOD statement for risk prediction models [15]. It was conducted at the San Cecilio University Hospital in Granada (Spain), with a reference population of almost 500,000 people.

### 2.2. Selection of Participants

This study included consecutive adult patients (age 18 years or older) by quota sampling, with infection confirmed by real-time reverse transcription–polymerase chain reaction (RT–PCR) to be SARS-CoV-2 positive [16], in the first 24 h of admission to the COVID-19 inpatient ward from 1 January 2021 to 20 March 2021.

Patients with suspected or confirmed bacterial pulmonary superinfection, those unable to cooperate with the examination (for physical or cognitive reasons), and those who voluntarily refused to participate were excluded from the study. All patients were given a study summary and signed an informed consent form with the possibility of revocation.

### 2.3. Measurements

Registration data, anthropometric variables, symptoms and onset date, comorbidities, vital signs, radiological severity, analytical data, and ultrasound lung involvement were compiled. The researchers on the COVID-19 transitional hospitalisation ward collected those clinical variables at the time of evaluation. The Emergency Department requested the analytical data at the first hospital contact, whereas the missing data were completed during the examination.

**Figure 1:**
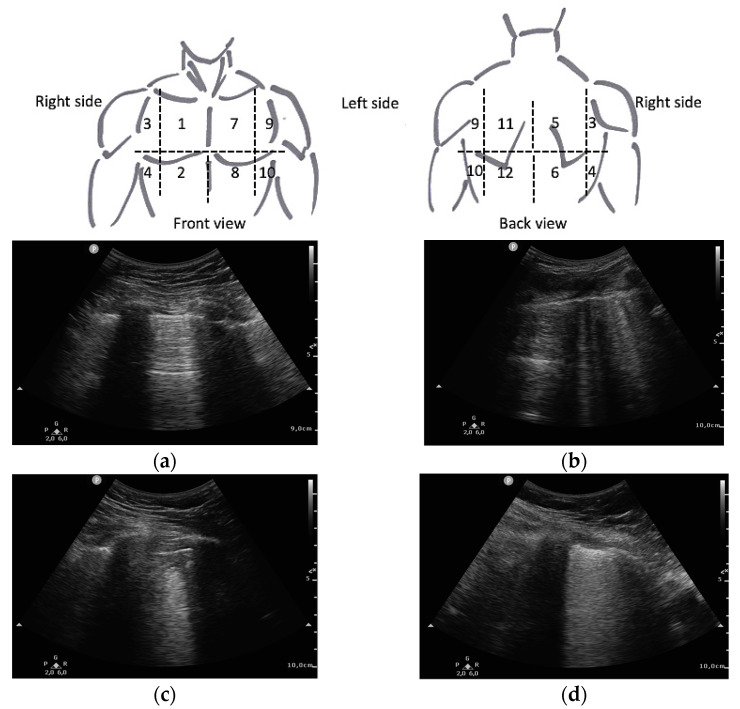
Lung areas distribution and LUS score as described by Soldati et al. [7]: (**a**) 0 points (normal A-lines pattern); (**b**) 1 point (>3 B-lines); (**c**) 2 points (subpleural consolidation); (**d**) 3 points (pulmonary consolidation or “with lung”).

Ultrasound imaging was performed with an ultrasound scanner PHILIPS^®^ SPARQ (Koninklijke Philips N. V.; Amsterdam, The Netherlands). The predefined lung programme was established, using a convex transducer 6–2 MHz. The Clinical Care Ultrasound Group of the hospital trained 6 Internal Medicine physicians in lung ultrasound, who obtained the images. At least 2 examiners were present when the ultrasound images were acquired in order to reduce the inherent inter-individual variability in this technique. We used a 12 lung fields protocol, as it is the protocol that has shown the best balance between accuracy and acquisition time [17]. The clavicular midline and paravertebral line divided each hemithorax into anterior, lateral, and posterior fields. These fields were further subdivided into superior and posterior fields, resulting in 6 fields in each lung (Figure 1). Prior examination of the patients medical history was avoided to prevent possible biases in image acquisition.

### 2.4. Outcomes

Outcomes collected were non-invasive mechanical ventilation or high-flow nasal oxygen therapy (NIMV), intubation (IMV), intensive care unit (ICU) admission, and death at 28 days. The combined outcome variables NIMV or IMV and NIMV or IMV or ICU admission or death at 28 days were also elaborated. Our primary variable was the combined variable poor outcome (NIMV or IMV or ICU admission or death at 28 days) and the secondary variable death at 28 days.

### 2.5. Scores Selection

The studied scores cover clinical, analytical, and imaging variables associated with poor prognosis in the evolution of COVID-19.

National Early Warning Score (NEWS) 2 [5], recognised in previous studies as a useful prognostic tool and superior to other scales in COVID 19 for both severe illness and in-hospital mortality [18], was used to analyse the prognostic value of clinical data. This takes into account vital signs, such as heart rate, systolic blood pressure, respiratory rate, peripheral oxygen saturation (SaO_2_), and body temperature; and it also includes the need for oxygen supply and the level of consciousness.

Furthermore, the most widely recognised international index for lung ultrasound in COVID-19 is the Lung Ultrasound Score (LUS) [11]. It scores from 0 to 3 each of the fields analysed, depending on the severity of the findings in each of them, as shown in Figure 1. The final sum of the score for all fields is used as a prognostic value. It was developed only correlating the score with clinical severity [7].

In addition to the scores mentioned above, two other indexes that include two or more of these categories were analysed. The COVID-19 Worsening Score (COWS) measures both LUS >15 points and clinical and analytical variables of the previously validated COVID-GRAM score [19], including dyspnea, symptom days, arterial oxygen pressure to oxygen inspired fraction ratio, and number of co-morbidities, including dementia, chronic obstructive pulmonary disease (COPD), chronic renal disease (CRD), diabetes mellitus (DM), hypertension, obesity, neoplasm, hepatitis B virus, cerebrovascular disease, cardiovascular disease, heart failure, and immunosuppression. The COWS measures the likelihood of developing severe disease in hospitalised patients [8]. Another prognostic tool is the COVID-19 Spanish Society of Infectious Diseases and Clinical Microbiology score (SEIMC Score), which studies clinical and analytical variables, such as age, low age-adjusted SaO_2_, neutrophil to lymphocyte ratio, estimated glomerular filtration rate, dyspnea, and sex in hospitalised patients and correlates it with 30-day mortality [9].

### 2.6. Analysis

Based on the data available at the beginning of the study, an estimated sample calculation of 22 patients was made to perform mean differences. We performed a simple logistic regression assuming an alpha of 0.05, a beta of 0.8, a probability of the event in the null hypothesis of 0.3, and a probability of the event in the alternative hypothesis of 0.7, with an estimated proportion of the population with the alternative hypothesis of 0.3. We set >15 as the severity cutoff point in the LUS, as used by COWS [8].

Descriptive statistics on the patients subjected to the ultrasonography exploration were performed by means and ranges for discrete variables, median, and inter-quartile ranges for continuous variables. For this analysis, normality was verified using the Shapiro–Wilk test. Non-normal variables means differences were tested using the Mann–Whitney test and the chi-square test for categorical variables. The main statistical analyses were derived in order to explore the relationship between our principal and second outcomes with several indices detailed above, such as NEWS2, LUS, COWS, or SEIMC Score. The methods consisted of univariate logistic regression models, estimating the risk of low index versus high index per unit depending on the classification. Receiver operating characteristic (ROC) curves were calculated to evaluate the predictive performance of these five scores for our primary and secondary outcomes. Areas under the curve (AUC) and 95% confidence intervals (CI) were also calculated. The cut-off point for the AUC was set at 0.7. This value indicates a favourable result, especially for the validation of a new score [20].

All tests were two-sided, and we determined a *p*-value < 0.05 and an AUC > 0.7 as significant. Data derivation procedure was calculated using SSPS software, version 24 (IBM Corp. Released 2017. IBM SPSS Statistics for Windows, Version 25.0. Armonk, NY, USA: IBM Corp.).

## 3. Results

### 3.1. Characteristics of Study Subjects

**Figure 2:**
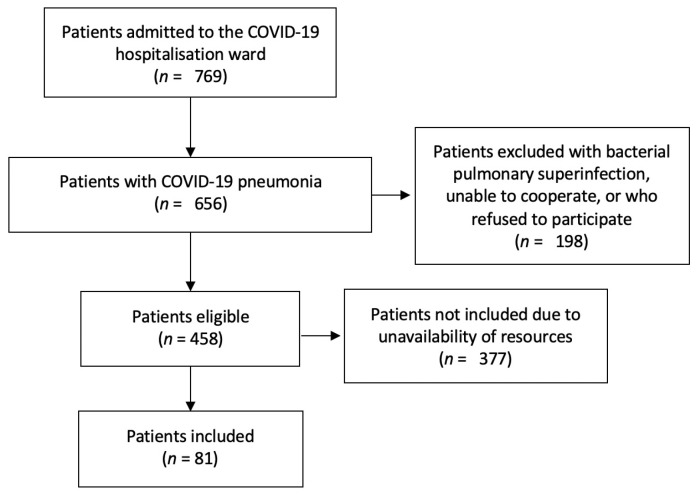
Patient selection flow chart.

Between 1 January and 20 March, a total of 769 patients were admitted to the COVID-19 hospitalisation ward of the San Cecilio University Hospital with a positive SARS-CoV-2 PCR, and 656 of those were admitted diagnosed of COVID-19 pneumonia. Of the remaining, 198 had one or more of the exclusion criteria described above. Finally, of the 458 eligible patients, 81 successive patients could be included in the study depending on the availability of human and material resources, as described in Figure 2.

The baseline characteristics of the participants are listed in Appendix A. The median age was 62 years (interquartile range 52–72), being significantly higher in dead patients (73) than in alive patients (59). The majority of participants were male (66.7%) although among the deceased, there was an equal number of males and females (4). Some comorbidities were more frequent in poor outcome and dead patients, such as COPD, CRD, immunosuppression (these three were statistically significant in death outcome), obesity, neoplasia, and heart failure. Radiologically, the median chest X-ray severity according to RALE was 4 (statistically significant for both main outcomes) and the incidence of pulmonary thromboembolism 8.6%, more frequent in favourable outcome (10% vs. 5%) and death (11.1% vs. 8.3%) without reaching statistical significance. In clinical variables, the median number of symptom days was higher in the living (8 vs. 5) and RR in poor outcome (26 vs. 21, *p* < 0.01), while in poor outcome and death, altered level of consciousness (5% vs. 3.3% and 22.2% vs. 1.3% with *p* < 0.01) and supplementary O_2_ (85% vs. 56.7% with *p* < 0.05 and 77.8% vs. 62.5%) were more frequent. Analytically, poor outcome and dead patients presented a higher frequency of levels above the established cut-off points in LDH and CRP scores although lower in D-dimer and neutrophil-lymphocyte ratio. They also had lower PaO_2_-FiO_2_ ratio and median eGFR (*p* < 0.01 in death outcome), lymphocyte, and platelet values (*p* < 0.05 in both outcomes) while higher NT-proBNP levels (*p* < 0.05 in death outcome).

### 3.2. Main Results

Differences between the subgroups for the two main outcomes under study are described in Table 1. In total, 21 patients in our cohort had a poor outcome, and nine patients died. Univariate analysis of each of the scores showed a statistically significant correlation between poor outcome and NEWS2 (OR 1.611, *p* < 0.001), LUS > 15 (OR 3.5, *p* = 0.019), and COWS (OR 3.968, *p* = 0.019). The study of death at 28 days showed statistically significant correlations in NEWS2 (OR 1.315, *p* = 0.041) and SEIMC Score (OR 1.190, *p* = 0.008).

**Figure 3:**
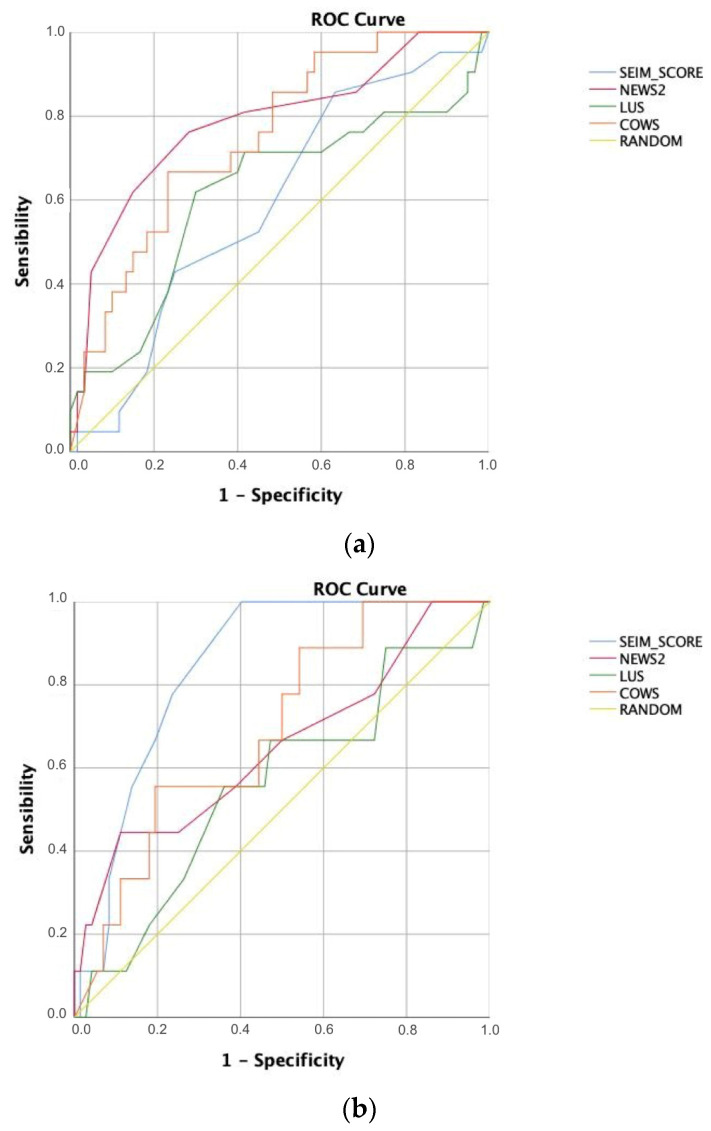
Pooled ROC curves for (**a**) poor outcome and (**b**) dead at 28 days.

The performance of each of the scores was represented in the pooled ROC curves (Figure 3), detailing their AUC, optimal cut-point value, sensitivity, and specificity in Table 2. As in the univariate analysis, NEWS (AUC 0.785), LUS > 15 (0.617), and COWS (AUC 0.751) showed the best performance at poor outcome. It also coincides in death at 28 days, with NEWS (AUC 0.654) and SEIMC Score (AUC 0.854) showing the best results although COWS (AUS 0.690) also performs well. Statistical differences between the AUC of the scores were also studied according to DeLong’s non-parametric method [21], as shown in Appendix B. As a result, NEWS (*p* = 0.028), SEIMC (*p* = 0.004), and COWS (*p* < 0.001) are superior to LUS as a continuous variable for predicting poor outcome, while COWS is also superior to LUS for predicting death at 28 days (*p* = 0.007). There is also significant superiority of NEWS over SEIMC for predicting poor outcome (*p* = 0.049).

## 4. Discussion

Most physicians agree on the importance of clinical findings of haemodynamic instability [22] and analytical findings of hyperinflammation syndrome [23] in the management of patients with COVID-19, supported by the classical radiological studies of chest X-ray and chest CT [24]. However, weighing each of these findings separately as a better predictor of the disease outcome proves to be very complicated. In this context, different scores that group several of these variables and assign a weight to them have emerged continuously up to the present time [25,26,27] as well as alternatives to conventional diagnostic tests, such as lung ultrasound [28]. However, a new question arises when trying to use these indices in clinical practice, where effectiveness in the management of time and resources is essential in a pandemic context. For this reason, we conducted this comparative study between representative scores of the most commonly used variables in medical practice (clinical, analytical, and imaging), avoiding some that have raised doubts about their usefulness [29].

Although several studies have attempted to deal with this problem, some of them have focused on validating previous indices not specific to COVID-19 [30,31], comparing them with new indices developed particularly for this disease [32,33] or comparing scores that evaluate one or two categories of the variables used to assess the severity of the disease [34]. However, none of them have validated COVID-specific indices taking into account the new diagnostic alternative of lung ultrasound or scores derived from it. More and more clinicians are becoming proficient in this technique, and it is no longer a diagnostic test offered by the radiology department but an increasingly accessible tool in the emergency department and on the hospital ward.

The results described above validate many of the original studies that developed the scores analysed. NEWS2 was confirmed as a useful tool both for predicting severe disease and for studying in-hospital death, showing significant results for both outcomes in our cohort. LUS findings had already shown good correlation with disease severity, repeating these results in this study with a cut-off point of 15. The SEIMC Score data for 30-day mortality were also confirmed, being significantly associated as well with 28-day death in our study. Finally, the COWS was developed to measure the probability of developing severe disease and, in our study, not only confirmed this result but also showed an acceptable diagnostic ability for 28-day mortality according to the AUC (however, it did not reach the pre-determined statistical significance), with a high sensitivity for both outcomes.

From the detailed analysis of the results, we appreciate the importance of the cut-off points. Setting the same cut-off point for the two prognostic variables (death and poor prognosis) may imply decreasing the utility, predicting one of the variables in favour of the other. In the case of LUS, on the other hand, it seems that its usefulness as a discrete variable is less than as an ordinal variable. Categorising into mild and severe groups with a cut-off point of 15 (as used by the COWS) has greater statistical significance in our sample than its AUC. The NEWS is a clinical scale, which measures the impact of COVID-19 at the time of the evaluation. Perhaps for this reason it is a better predictor of poor outcome, as clinical variables are established criteria for starting mechanical ventilation. However, its usefulness in predicting death as an outcome is less in the AUC analysis although it is possible that with greater sample power it would reach statistical significance, as it does in simple logistic regression. The COWS has a similar issue, in which a larger sample size would possibly show statistical significance in the analysis of death as an outcome (AUC 0.69, having used 0.7 as the cut-off point for statistical significance). Finally, the SEIMC Score appears to be an index developed specifically to predict death as an outcome, including the variables that, in the cohort analysed, best predicted this outcome. Specifically, the inclusion of age and eGFR as variables in their calculation may be two of the reasons for this. Our study provides external validity to these results but confirms that it is not a useful score for predicting poor prognosis.

Among the strengths of our study, as an underused clinical tool equal to other more commonly used techniques such as blood tests, it is worth noting the inclusion of lung ultrasound and the scores derived from it. Finally, the outcomes measured included both poor outcome and death at 28 days, in contrast to other studies that assessed only one or the other, and there were been any missing data among participants.

The study was designed with the purpose to reduce the limitation of being an operator-dependent technique, with two or more operators always required to be present during image acquisition and interpretation. However, the biases in lung ultrasound performance by our team of operators, although mitigated by the presence of other colleagues, were still present and cannot be completely ruled out. Potential limitations of the study could arise from the sample size. Although the initial calculation showed us that the sample of 24 patients was sufficient, probably with a larger sample, we could perform a mutivariate analysis, including joint clinical and ultrasound parameters, and confirm the clear tendency of some variables to reach statistical significance without reaching it. Participants included among the eligible patients were recruited on a randomised basis, so we believe that the patients included are a representative sample of the total population admitted to the inpatient ward of our hospital and therefore do not represent a bias in itself. In addition, some parameters, such as D-dimer or cardiovascular disease, are more frequent in favourable outcomes than in other studies, which could probably change with more cases favouring regression to the mean. Though a careful selection was made from among the many existing scores, selecting representative examples of the variables most commonly used in clinical practice, the continuous proliferation of these indices makes it impossible to compare all of them. At last, the exclusion of patients with potential bacterial superinfection in the lung may be a bias that eliminates patients with more severe evolution, while the inclusion of lung ultrasound implies an operator-dependent technique that may make reproducibility in other studies difficult.

This study raises some issues that will need to be addressed in further studies. Which type of transducer (convex or linear) is most suitable for lung ultrasound should be defined and demonstrated. Most of these scores have also been developed and validated in the hospitalised population, and studies are needed to test their usefulness in the out-of-hospital setting. Our study indicates the necessity of establishing LUS cut-off points for mild, moderate, and severe disease in future studies. We also consider it necessary to establish guidelines in the elaboration of these scores and to combine those that measure the same variables into indices with greater statistical power.

## 5. Conclusions

In summary, NEWS2, LUS, and COWS are good scores for predicting poor outcome of COVID-19, while NEWS2, SEIMC Score, and COWS are useful scores for predicting death at 28 days. Both COWS and NEWS are the scores that best predict the evolution of COVID patients in our cohort. Lung ultrasound is a diagnostic tool that should be included in the evaluation of COVID-19 patients.

## Figures and Tables

**Table 1:** Differences between favourable and poor outcome and between alive and dead patients.

	Vs. Poor Outcome		Vs. Dead at 28 Days	
	*p*	OR (CI)	*p*	OR (CI)
**NEWS2**	0.001 **	1.611 (1.228–2.113)	0.041 *	1.315 (1.012–1.710)
**LUS > 15**	0.019 *	3.5 (1.192–10.275)	0.271	2.235 (0.519–9.636)
**SEIMC Score**	0.516	1.034 (0.935–1.143)	0.008 **	1.190 (1.046–1.354)
**COWS**	0.019 *	3.968 (1.251–12.573)	0.243	2.552 (0.530–12.283)

* *p* < 0.05; ** *p* < 0.01.

**Table 2:** Performance of prognostic scores for (**a**) poor outcome and (**b**) dead at 28 days.

(a)				
Poor Outcome	AUC	Optimal Cut-Off Point Value	Sensitivity	Specificity
**NEWS2**	0.785 *	>5	0.619	0.850
**LUS**	0.617	>17	0.619	0.700
**SEIMC Score**	0.593	>9	0.333	0.783
**COWS**	0.751 *	≥0.1007	0.857	0.617
**(b)**				
**Dead 28 Days**	**AUC**	**Optimal Cut-Off Point Value**	**Sensitivity**	**Specificity**
**NEWS2**	0.654	>5	0.444	0.887
**LUS**	0.560	>17	0.556	0.634
**SEIMC Score**	0.840 *	>9	0.667	0.803
**COWS**	0.690	≥0.1007	0.889	0.431

* AUC > 0.7.

## Data Availability

Not applicable.

## Appendix A

**Table A1:** Clinical, analytical and imaging characteristics of participants.

	All Patients (*n* = 81)	Poor Outcome (*n* = 21)	Favourable Outcome (*n* = 60)	*p*-Value	Dead 28 Days (*n* = 9)	Alive 28 Days (*n* = 72)	*p*-Value
**Age**	62	61	62	0.550	73	59	0.001 **
(median, IQR)	(52–72)	(47.5–72.3)	(52.8–71.3)		(72–78)	(51–70)	
**Sex**							
Male (%)	66.7	75	63.9	0.362	55.6	68.1	
Female (%)	33.3	25	36.1		44.4	31.9	0.292
**Weight** [kg]	84	84	84	0.852	83.9	84	0.733
(mean, SD)	(1.9)	(3.3)	(2.3)		(7.3)	(1.9)	
**Height** [cm]	168.7	168	168.9	0.653	165	169	0.868
(mean, SD)	(1.1)	(2.1)	(1.3)		(3.7)	(1.2)	
**Dementia** (%)	2.5	0	3.3	0.412	0	2.7	0.635
**COPD** ^1^ (%)	4.9	10	3.3	0.229	33.3	1.4	0.001 **
**Chronic renal disease** (%)	12.4	15	11.7	0.678	33.3	9.7	0.023 *
**Diabetes mellitus** (%)	28.4	25	30	0.698	55.6	25	0.153
**Hypertension** (%)	46.9	30	53.3	0.081	55.6	45.8	0.854
**Obesity** (%)	34.6	40	33.3	0.600	44.4	33.3	0.505
**Neoplasia** (%)	15.2	20	13.6	0.414	33.3	12.9	0.064
**Hepatitis B virus** (%)	0	0	0	-	0	0	-
**Cerebrovascular disease** (%)	5.1	5	5.1	0.964	0	5.7	0.491
**Cardiovascular disease** (%)	8.9	0	11.9	0.119	0	10	0.352
**Heart failure** (%)	7.6	15	5.1	0.122	22.2	5.7	0.050
**Immunosuppression** (%)	6.4	10	5.1	0.400	22.2	4.3	0.023 *
**Number of comorbidities**	1	1	1	0.402	2	1	0.006 **
(median, IQR)	(0–2)	(0–2)	(0–2)		(2–3)	(0–2)	
**Days of symptoms**	8	7	9	0.071	5	8	0.057
(median, IQR)	(5–10)	(4.75–9)	(6–10)		(4–6)	(7–10)	
**Dyspnea** (%)	69.1	75	66.7	0.513	66.7	69.4	0.705
**Cough** (%)	76.5	75	76.7	0.851	66.7	77.8	0.914
**Altered consciousness** (%)	3.7	5	3.3	0.724	22.2	1.3	0.001 **
**Fever** (%)	44.4	50	41.7	0.466	44.4	44.4	0.598
**SaO_2_** ^2^ [%]	96.2	95.2	96.6	0.012 *	95	96.4	0.014 *
(mean, SD)	(0.2)	(0.6)	(0.2)		(0.9)	(0.24)	
**Supplementary O_2_** (%)	64.2	85	56.7	0.025 *	77.8	62.5	0.502
**RR** ^3^ [breaths/min]	24	26	21	0.009 **	24	24	0.245
(median, IQR)	(20–28)	(24–28)	(18–24.5)		(22–28)	(21–28)	
**HR** ^4^ [beats/min]	81	85	76	0.030 *	82	81	0.692
(median, IQR)	(70–90)	(79.5–96)	(67.8–89)		(73–86)	(70–90)	
**SBP** ^5^ **(mmHg)**	127.3	127.5	127.2	0.939	130.9	126.8	0.893
(mean, SD)	(1.7)	(2.9)	(2.1)		(7.3)	(1.7)	
**Rx. thorax** [RALE ^6^]	4	4	4	0.019 *	5	4	0.001 **
(median, IQR)	(3–5)	(3–5)	(3–5)		(5–8)	(3–5)	
**CT PTE** ^7^ (%)	8,6	5	10,0	0.504	11.1	8.3	0.682
**PaO_2_-FiO_2_** ^8^ **ratio**	366.3	306.2	388.3	0.001 **	289.9	373.1	0.015 *
(mean, SD)	(10.8)	(23)	(11.1)		(30.8)	(10.8)	
**Glucose** [mg/dL]	147.2	126.75	154	0.139	160.7	145.5	0.862
(mean, SD)	(7.5)	(9.5)	(9.4)		(22.2)	(8)	
**eGFR** ^9^							0.001 **
[ml/min/1.73 m^2 9^]	80	75	81	0.330	52.6	83.4	
(mean, SD)	(2.8)	(5.9)	(3.1)		(7.4)	(2.7)	
**AST** ^10^ [U/L]	52.6	66	48.6	0.920	39.2	47.6	0.837
(mean, SD)	(6.3)	(17.7)	(6.1)		(6.7)	(6.5)	
**Ferritin** [ng/mL]	824.6	745.8	848.4		681.5	832.5	
(mean, SD)	(80.7)	(132.7)	(99.3)	0.814	(183.3)	(87.7)	0.914
>700 ng/mL (%)	44.4	45	43.3	0.852	33.3	45.2	0.731
**LDH** ^11^ [U/L]	337.2	365.9	329.6	0.738	339.8	336.9	0.849
(mean, SD)	(13.3)	(33.4)	(14)	0.254	(44.9)	(14)	0.769
>400 U/L (%)	21	30	18.3		22.2	20.8	
**CRP** ^12^ [mg/L]	91.5	84.2	92.4	0.473	91.5	91.5	0.681
(mean, SD)	(6.8)	(14.1)	(7.7)		(23)	(7.1)	
>15 mg/mL (%)	95.1	100	93.3	0.240	100	94.4	0.497
**Lymphocyte** [10^3^/μL]	0.87	0.83	0.88	0.763	0.7	0.89	0.101
(mean, SD)	(0.05)	(0.1)	(0,06)		(0.15)	(0.05)	
**Neutrophil-lymphocyte ratio**	6.4	7.3	6.1	0.540	8.6	6.1	0.264
(mean, SD)	(0.46)	(0.8)	(0.6)	0.711	(2.1)	(0.4)	0.706
>10 (%)	17.3	7.31	16.7		11.1	18.1	
**Platelets** [10^3^/μL]	200.6	163.4	214	0.008 **	152.6	206.6	0.020 *
(mean, SD)	(8.3)	(12.7)	(9.9)		(20.6)	(8.8)	
**D dimer** [mg/L]	1.68	1.1	1.9	0.144	4.4	1.3	0.168
(mean, SD)	(0.5)	(0.3)	(0.6)	0.538	(3.9)	(0.2)	0.587
>15 mg/mL (%)	19.7	10	23.3		11.1	20.8	
**NT-proBNP** ^13^ [pg/mL]	815	1007.7	741.8	0.180	2086.9	593.8	0.038 *
(mean, SD)	(171.2)	(372.5)	(188.7)		(731.9)	(136.6)	
**NEWS2**	4	6	3	0.001 **	5	3	0.041 *
(median, IQR)	(2–6)	(5–7)	(2–5)		(3–7)	(2–6)	
**ECO LUS total**	15	18	14	0.177	18	15	0.541
(median, IQR)	(10–19)	(13.5–19)	(9.8–18.3)	0.019 *	(10–19)	(10–19)	0.271
>15 (%)	49.4	70	43.3		66.7	47,2	
**SEIMC Score**	6	6	6	0.516	11	5	0.008 **
(median, IQR)	(4–10)	(5–10)	(4–9)		(9–14)	(4–8)	
**COWS**	0.52	0.72	0.47	0.019 *	0.71	0.5	0.243
(mean, SD)	(0.05)	(0.10)	(0.06)		(0.15)	(0.06)	

^1^ COPD: Chronic obstructive pulmonary disease; ^2^ SaO_2_: Peripheral arterial oxygen saturation; ^3^ RR: Respiration rate; ^4^ HR: Heart rate; ^5^ SBP: Systolic blood pressure; ^6^ RALE: Radiographic Assessment of Lung (O)Edema; ^7^ CT PTE: Computer Tomography Pulmonary Thromboembolism; ^8^ PaO_2_-FiO_2_: Arterial oxygen pressure to oxygen inspired fraction ratio; ^9^ eGFR: Estimated glomerular filtration rate calculated by the CKD-EPI; ^10^ AST: Aspartate aminotransferase; ^11^ LDH: Lactate dehydrogenase; ^12^ CRP: C-reactive protein; ^13^ NT-proBNP: N-terminal pro-brain natriuretic peptide. (* *p* < 0.05 and ** *p* < 0.01).

## Appendix B

**Table A2:** Statistical differences between the AUC of the scores according to DeLong’s non-parametric method [21].

	Poor Outcome *p*-Value (CI)	Dead 28 Days *p*-Value (CI)
**LUS vs. NEWS2**	0.028 * (−0.319–0.018)	0.507 (−0.357–0.176)
**LUS vs. SEIMC**	0.004 ** (−0.471–0.090)	0.812 (−0.172–0.220)
**LUS vs. COWS**	< 0.001 ** (−0.201–0.067)	0.007 ** (−0.226–0.036)
**COWS vs. NEWS2**	0.590 (−0.160–0.233)	0.676 (−0.151–0.091)
**COWS vs. SEIMC**	0.059 (−0.006–0.322)	0.081 (−0.318–0.018)
**NEWS2 vs. SEIMC**	0.049 * (0.0003–0.385)	0.126 (−0.435–0.054)

* *p* < 0.05; ** *p* < 0.01.
